# Hyperspectral imaging for estimating leaf, flower, and fruit macronutrient concentrations and predicting strawberry yields

**DOI:** 10.1007/s11356-023-30344-8

**Published:** 2023-10-19

**Authors:** Cao Dinh Dung, Stephen J. Trueman, Helen M. Wallace, Michael B. Farrar, Tsvakai Gama, Iman Tahmasbian, Shahla Hosseini Bai

**Affiliations:** 1 Centre for Bioinnovation, University of the Sunshine Coast, 90 Sippy Downs Drive, Sippy Downs, QLD 4556 Australia; 2 School of Science, Technology and Engineering, University of the Sunshine Coast, 90 Sippy Downs Drive, Sippy Downs, QLD 4556 Australia; 3 Potato, Vegetable and Flower Research Center – Institute of Agricultural Science for Southern Vietnam, Thai Phien Village, Ward 12, Da Lat, Lam Dong Vietnam; 4 Centre for Planetary Health and Food Security, School of Environment and Science, Griffith University, Nathan, Brisbane, QLD 4111 Australia; 5 Department of Agriculture and Fisheries, Queensland Government, Toowoomba, QLD 4350 Australia

**Keywords:** Calcium, Hyperspectral imaging, Nitrogen, Potassium, Phosphorus

## Abstract

Managing the nutritional status of strawberry plants is critical for optimizing yield. This study evaluated the potential of hyperspectral imaging (400–1,000 nm) to estimate nitrogen (N), phosphorus (P), potassium (K), and calcium (Ca) concentrations in strawberry leaves, flowers, unripe fruit, and ripe fruit and to predict plant yield. Partial least squares regression (PLSR) models were developed to estimate nutrient concentrations. The determination coefficient of prediction (*R*^2^_*P*_) and ratio of performance to deviation (RPD) were used to evaluate prediction accuracy, which often proved to be greater for leaves, flowers, and unripe fruit than for ripe fruit. The prediction accuracies for N concentration were *R*^2^_*P*_ = 0.64, 0.60, 0.81, and 0.30, and RPD = 1.64, 1.59, 2.64, and 1.31, for leaves, flowers, unripe fruit, and ripe fruit, respectively. Prediction accuracies for Ca concentrations were *R*^2^_*P*_ = 0.70, 0.62, 0.61, and 0.03, and RPD = 1.77, 1.63, 1.60, and 1.15, for the same respective plant parts. Yield and fruit mass only had significant linear relationships with the Difference Vegetation Index (*R*^2^ = 0.256 and 0.266, respectively) among the eleven vegetation indices tested. Hyperspectral imaging showed potential for estimating nutrient status in strawberry crops. This technology will assist growers to make rapid nutrient-management decisions, allowing for optimal yield and quality.

**Supplementary Information:**

The online version contains supplementary material available at 10.1007/s11356-023-30344-8.

## Introduction

Global demand for food is increasing due to the continued rise in the world population (Godfray et al. 2010). Fertilizers play an important role in maximizing food production but fruit, nut, and seed yields are often sustained by excessive fertilizer use (Islam et al. 2022; Trejo-Téllez and Gómez-Merino 2014). Non-optimal fertilizer applications cause negative impacts on plant growth and development (Kulkarni and Goswami 2019). Over-fertilization, for example, can reduce yield and have negative impacts on the environment, while insufficient fertilization can also reduce yield and decrease product quality (González et al. 2015; Hapuarachchi et al. 2022; Pereira et al. 2015; Trejo-Téllez and Gómez-Merino 2014). Farmers often make decisions on nutrient amendments without full knowledge of the nutrient status of their crop (Bindraban et al. 2015; Islam et al 2022; Sheriff 2005) because current methods to examine plant nutrient status are laborious, costly, and time consuming (Yanli et al. 2015). Knowledge of plant nutrient status in real time would allow timely decisions on how much fertilizer needs to be added to a crop.

Hyperspectral imaging has been applied widely in agriculture, food, medicine, and other fields to estimate the internal qualities of scanned objects (Bai et al. 2018; Davur et al. 2023; ElMasry et al. 2012; Farrar et al. 2023; Gowen et al. 2007; Han et al. 2021; Huang et al. 2014; Malmir et al. 2020; Moscetti et al. 2015). Hyperspectral imaging combines spectroscopy with imaging techniques to acquire both spectral and spatial information simultaneously (ElMasry et al. 2012; Huang et al. 2014). Hyperspectral imaging is potentially non-destructive, low-cost, and reliable and has been applied to fruit, nuts, grains, and vegetables to estimate internal qualities such as total soluble solid concentration and moisture content, as well as firmness, ripeness, and shelf life (Bai et al. 2018; Davur et al. 2023; Gómez et al. 2006; Han et al. 2021; Han et al. 2023; Peng and Lu 2008; Pérez-Marín et al. 2009; Rajkumar et al. 2012; Ravikanth et al. 2017). Hyperspectral imaging allows the estimation of mineral nutrient concentrations such as nitrogen (N), phosphorus (P), potassium (K), and calcium (Ca) in the soil and leaves of many crops (Ferwerda et al. 2005; Mahajan et al. 2017; Pacumbaba and Beyl 2011; Pandey et al. 2017; Rodriguez et al. 2006; Tahmasbian et al. 2018; Yu et al. 2014). Hyperspectral imaging can also be used to estimate the concentrations of mineral nutrients including N, P, K, and Ca in avocado fruit (Kämper et al. 2020). Hyperspectral images obtained from the canopy or leaves have also been used to predict crop yield (Aparicio et al. 2000; Babar et al. 2006; Cao et al. 2015; Prasad et al. 2007; Xie et al. 2020). For example, grain yields have been predicted using vegetation indices that are based on canopy hyperspectral imaging (Cao et al. 2015).

Strawberry is a valuable fruit crop that is produced in many countries (FAOSTAT 2021). Strawberry fruit are beneficial for human health because of their high nutrient and antioxidant concentrations (Giampieri et al. 2012; Hannum 2004; Mahmood et al. 2012). Strawberry yield and fruit quality, e.g., fruit size, sweetness, firmness, and shelf life, are influenced by the concentrations of macronutrients such as N, P, K, and Ca in leaves (Chen et al. 2011; Nestby et al. 2005; Trejo-Téllez and Gómez-Merino 2014). Hyperspectral imaging that encompasses visible and near infrared wavelengths has been used to estimate total soluble solid concentration, acid concentration, and moisture content of strawberry fruit (ElMasry et al. 2007; Nagata et al. 2004; Shao and He 2007). Nitrogen concentrations in the strawberry canopy have been estimated with a portable field spectroradiometer (350–1,050 nm) (España-Boquera et al. 2006). However, hyperspectral imaging using visible and near infrared wavelengths has not been tested for its potential to estimate N, P, K, and Ca concentrations in strawberry leaves, flowers, unripe fruit, and ripe fruit or to predict strawberry fruit yield.

Machine vision technologies, including mobile hyperspectral cameras developed for use on farms, are usually complicated and not easily operated by farm managers (Tian et al. 2020). Hence, most machine vision technologies developed for agricultural systems remain underutilized. Laboratory-based hyperspectral imaging that is easy to operate could be a breakthrough for adapting the technology to plant nutrient assessment (Farrar et al. 2021; Malmir et al. 2020). We aimed specifically to determine the accuracy of hyperspectral imaging to estimate N, P, K, and Ca concentrations in strawberry leaves, flowers, unripe fruit, and ripe fruit. We also aimed to evaluate the potential of hyperspectral imaging to predict strawberry fruit yield and one of its main components, fruit mass.

## Materials and methods

### Plant samples

We established 100 pots, each pot containing one rooted runner, for undertaking a series of experiments to understand the effects of pollination on fruit quality, shelf life, and yield under different levels of calcium nutrition (Dung et al. 2021, 2022, 2023). We used the subtropical strawberry cultivar, Redlands Joy. The plants had been established from rooted runners that were transplanted in May 2018 into 4.5 L pots containing coco-peat (EC < 1 mS/cm, pH = 5.5–7.0) and perlite (4:1, v:v) with 2.5 g of Osmocote fertilizer (N:P:K = 19.6:16.0:5.0% w/w, plus trace elements) (Scotts International, Heerlen, The Netherlands). The potted plants were placed in a glasshouse at the University of the Sunshine Coast, Sippy Downs, Australia (26° 43′ S 153° 03′ E). The daily temperature and photosynthetic photon flux density in the glasshouse were described previously (Dung et al. 2021). We top-dressed each plant monthly with 15 g of Osmocote fertilizer and applied a supplementary 5 mL of 1% (v/v) aqueous PowerFeed® foliar fertilizer (Seasol International, Bayswater, Australia) weekly during the first 10 weeks from transplanting. Sixty of the 100 plants were then sprayed fortnightly with eight sprays of Ca as Grotek Cal-Max (GS Distribution, Langley, Canada) at either 1, 2, or 4 kg elemental Ca ha^−1^ spray^−1^. Each plant received approximately 5 mL of solution at each spray. The other 40 plants received no Ca sprays. Water was manually supplied daily to plants, with approximately 150 mL applied per potted plant.

### Sample collection and preparation

**Fig. 1:**
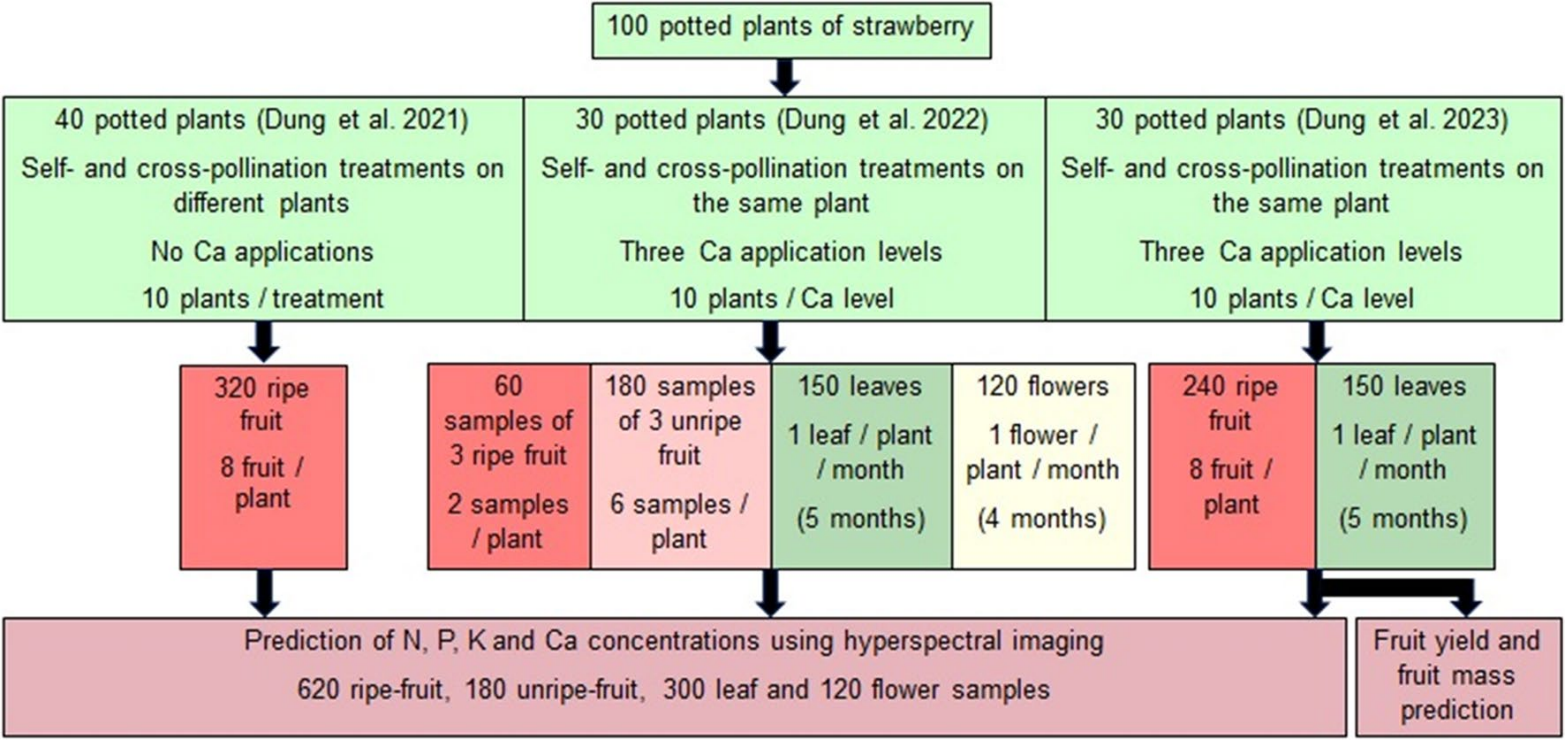
Summary of strawberry fruit, leaf, and flower samples for prediction of nutrient concentrations, fruit yield, and fruit mass

**Fig. 2:**
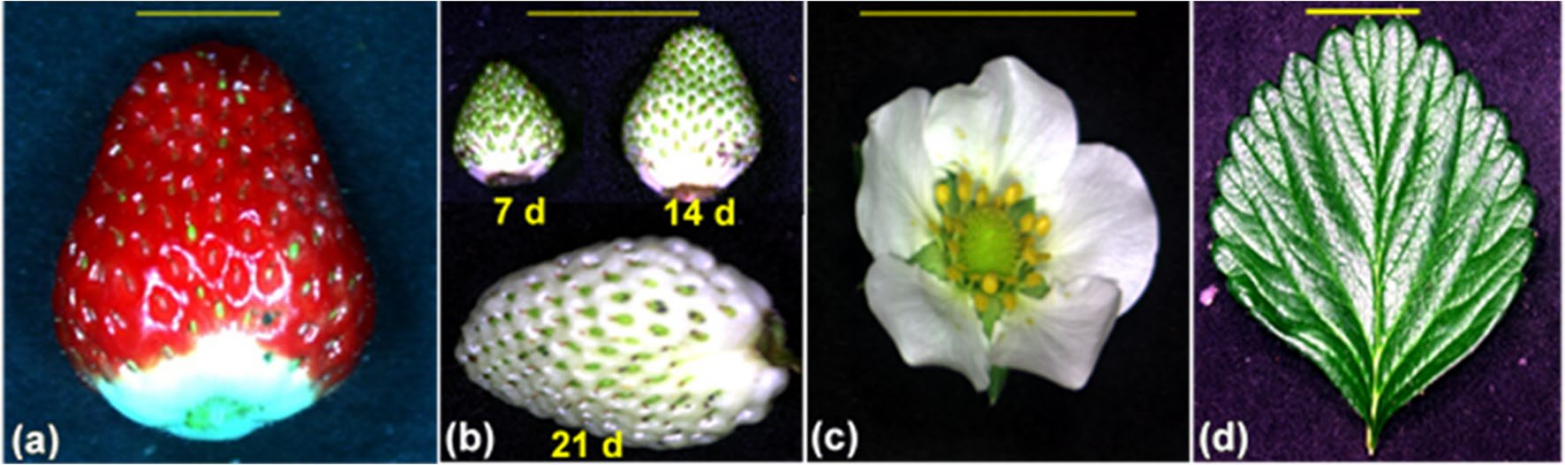
Strawberry **a** ripe fruit; **b** unripe fruit harvested at 7, 14, or 21 d after first pollination; **c** flower; and **d** leaf used for developing models to estimate nitrogen, phosphorus, potassium, and calcium concentrations. Scale bars = 1 cm

We collected fruit, leaf, and flower samples from July to October 2018 as described previously by Dung et al. (2021, 2022, 2023). All 100 plants were used for nutrient prediction in leaves and fruit, 30 plants were used for nutrient prediction in flowers, and 30 plants were used for yield and fruit-mass prediction (Figs. 1 and 2). In brief, ripe fruit were collected from 40 plants that did not receive Ca sprays and 60 plants that received Ca sprays. Unripe fruit were harvested at 7, 14, and 21 d after pollination from 30 plants that received Ca sprays. We harvested five leaves per plant when the leaves opened fully, with each of the five leaves being sampled at monthly intervals over 5 months from 60 plants that received Ca sprays. We harvested four flowers (with sepals) per plant when the flowers opened fully from 30 plants in the third study, with each of the four flowers being sampled at monthly intervals over 4 months. Harvested samples were transferred immediately to the laboratory for hyperspectral imaging. The fresh leaves and flowers were imaged, dried at 70 °C for 24 h, and then used for N, P, K, and Ca analyses. The unripe fruit and ripe fruit were imaged without sepals and pedicels, stored fresh at − 20 °C, and then also used for N, P, K, and Ca analyses. The total number of samples included 620 ripe-fruit samples, 180 unripe-fruit samples, 300 leaf samples, and 120 flower samples (Fig. 1).

### Hyperspectral imaging

We used a laboratory-based hyperspectral imaging system (Benchtop System, Resonon, Bozeman, MT) with a 12-bit line scanner camera (Pika XC2, Bozeman, MT) to capture images (Farrar et al. 2021). The system comprised a hyperspectral camera with a 23-mm focal-length lens, a linear transition stage operated by a stepper motor, four current-controlled wide-spectrum quartz-halogen lights, and a main-control computer (Fig. A1).

We placed the samples on a black background on the transition stage of the camera. Images were captured using a visible/near-infrared hyperspectral imaging system in the spectral range of 400–1,000 nm. The spectral resolution was 1.3 nm, producing a total of 462 grayscale images that can be generated to form 462 bands of each hyperspectral image. The exposure time was adjusted to 27.3 ms and the stage moved at 1.23 mm s^−1^.

SpectrononPro software package (Version 2.94, Resonon, Bozeman, MT) was used to extract reflectance data (spectral information) of the acquired leaf, flower, unripe, and ripe fruit images. The mean corrected relative reflectance (*R*) was calculated from the raw spectral reflectance, *R*_0_, within SpectrononPro as shown in Eq. 1 (ElMasry et al. 2007):

1 $$R=\frac{{R}_{0}-D}{W-D}$$

where *R*_0_ was the raw spectral reflectance, *D* was the reflectance of a dark image (camera lens covered), and *W* was the reflectance of a white Teflon board that reflected approximately 99% of incident light. This corrected for the spectral curve of the leaf, flower, or fruit surface. The 100% reflectivity was scaled to 10,000 (integers) by default. The mean corrected relative reflectance was used for model development.

### Nutrient analysis

Nitrogen concentrations of leaf, flower, unripe-fruit, and ripe-fruit samples were determined by combustion analysis using a LECO 928 analyzer (LECO, Saint Joseph, MI) (McGeehan and Naylor 1988; Muñoz-Huerta et al. 2013; Rayment and Higginson 1992). Calcium, phosphorus, and potassium concentrations were analyzed by using inductively coupled plasma–atomic emission spectroscopy on samples that were open-vessel digested with a 5:1 mixture of nitric and perchloric acids (Munter and Grande 1980).

### Data analysis and model development

Image acquisition and data extraction were conducted using SpectrononPro software (Version 2.94, Resonon, Bozeman, MT). The mean raw reflectance was extracted by marking a region of interest (ROI) for each image. The ROI for leaf, flower, unripe-fruit, and ripe-fruit images contained the surface of one side of the leaf, flower, unripe fruit, or ripe fruit, respectively (Fig. A2). We mixed three fruit together for nutrient analysis in the case of composite fruit samples and so hyperspectral data from these three fruit were averaged prior to data analysis and model development. Spectral outliers in the samples, if any, were detected using a Hotelling’s *T*^2^ test (with 95% level of confidence) and removed from the data set (Farrar et al. 2021). The remaining data were divided randomly into two data sets, one used for calibration (80%) and the other (20%) used as a test data set to examine the precision of prediction using developed models (Table A1). We used a leave-one-out (full) cross-validation to evaluate the performance of the model (Dai et al. 2014; Tahmasbian et al. 2017; Zhang et al. 2013). This method uses the calibration data set but leaves one sample out of the calibration set each time and assesses the model using the remaining data. In the next iteration, another sample is left out randomly for the validation and this process continues until every sample is left out of the model once (Dai et al. 2014; Tahmasbian et al. 2017; Zhang et al. 2013).

We developed partial least square regression (PLSR) models. PLSR is one of the most frequently used modeling methods applied in hyperspectral imaging studies (De Silva et al. 2023; Han et al. 2023; Kämper et al. 2020; Mayr et al. 2021). The PLSR is commonly recommended when the dataset is small (Wold et al. 2001a). The PLSR is a linear multivariate model and relates data matrices of *X* and *Y*, the predicted and observable variables, respectively, by identifying smaller sets of predictors to perform a series of regressions (Wold et al. 2001a). These predictors have linear combinations, and the model is able to analyze datasets with noise and incomplete variables (Wold et al. 2001b). We also applied spectral pre-processing methods to decrease noise and improve model performance (Qin et al. 2013). The applied pre-processing methods were Smoothing Savitzky-Golay (Smoothing S-Golay, 1st derivative), Normalize, Derivative Savitzky-Golay (Derivative S-Golay), and Standard Normal Variate (SNV) (Qin et al. 2013). Developed models were assessed using the following indices: determination coefficients of calibration (*R*^2^_*C*_), validation (*R*^2^_*V*_), and prediction (*R*^2^_*P*_); root mean squares error (RMS_E_) of calibration (RMSE_C_), validation (RMSE_V_), and prediction (RMSE_P_); and ratio of performance to deviation (RPD). Only the model with highest coefficient of determination (*R*^2^), ratio of performance to deviation (RPD), and lowest root mean squares error (RMSE) was selected to determine the accuracy of estimation for each targeted nutrient (Table 1). The *R*^2^ and RMSE were calculated using Eqs. 2 and 3 (Yanli et al. 2015):

2 $${R}^{2}=1- \frac{{\sum }_{i=1}^{n}{\left({y}_{\mathrm{i}}-{\widehat{y}}_{\mathrm{i}}\right)}^{2}}{{\sum }_{i=1}^{n}{\left({y}_{\mathrm{i}}- \overline{y }\right)}^{2}}$$

3 $$\mathrm{RMSE}=\sqrt{\frac{\sum_{n=1 }^{n}{\left({\widehat{y}}_{\mathrm{i}} -{y}_{\mathrm{i}}\right)}^{2}}{n}}$$

where *n* indicated the number of samples, *y*_*i*_ and *ŷ*_*i*_ represented the reference and predicted values of the *i*th sample, respectively, and *ȳ* represented the mean of each reference value.

We then calculated the RPD using the test set (Farrar et al. 2021; Morellos et al. 2016). The RPD indicates the appropriateness of prediction. RPD classifications include RPD of 1.5–2.0 discriminates between high and low values, RPD of 2.0–2.5 provides a coarse quantitative prediction, and RPD > 2.5 and RPD > 3.0 provide good and excellent predictions, respectively (Nicolaï et al. 2007). The higher the RPD, the more robust is the model (Farrar et al. 2021; Kamruzzaman et al. 2012). RPD was defined using Eq. 4:

4 $$\mathrm{RPD}={\mathrm{SD}}_{\mathrm{TEST}}/{\mathrm{RMSE}}_{\mathrm{TEST}}$$

where SD_TEST_ was the standard deviation of the observed values and RMSE_TEST_ was the root mean square error of the prediction from the test set.

We identified specific wavelengths that were important for predicting N, P, K, and Ca concentrations using β-coefficient values that carry predictive information. The wavelengths with the highest β-coefficients contribute most to the predictive ability of the models (Iqbal et al. 2013; Malmir et al. 2019; Tahmasbian et al. 2021; Xu et al. 2018). We assessed the model accuracies in predicting nutrient concentrations of fruit, leaves, and flowers by comparing the best-fit model for each plant part, with this model being the one that had the highest RPD and *R*^2^ of the dataset. Unscrambler® X software version 11 (CAMO Software Inc., Trondheim, Norway) was used for all computation, spectral data transformations, PLSR computations, outlier detection, and model development (Farrar et al. 2021).

### Predicting yield and fruit mass

We attempted to predict both fruit yield and fruit mass using hyperspectral images. We also attempted to predict fruit yield and fruit mass from the macronutrient concentrations in leaf samples (*n* = 150) (Fig. 1). Yield was calculated as the total mass of fruit harvested from each plant during the study period of July to October 2018. Hyperspectral data from leaves, nutrient concentrations of leaves, and fruit mass were averaged for each plant, while yield was calculated as total fruit mass per plant. Stepwise regression was performed to evaluate linear regressions between the concentrations of N, P, K, and Ca in leaves or vegetation indices as the independent variable and fruit mass or yield as the dependent variable. Vegetation indices used to predict yield were Difference Vegetation Index (DVI), Modified Chlorophyll Absorption Ratio Index (MCARI), Modified Triangle Vegetation Index (MTVI), Normalized Difference Vegetation Index (NDVI), Photochemical Reflectance Index (PRI), Enhanced Vegetation Index (EVI), Ratio Vegetation Index (RVI), Infrared Percentage Vegetation Index (IPVI), Structure Independent Pigments Index (SIPI), and Red Edge Vegetation Stress Index (RVSI) (Rathod et al. 2013; Wang et al. 2018; Yu et al. 2018). The equations for all indices are presented in Table A2. Linear regressions were regarded as significant at *p* < 0.05.

## Results

### Descriptive statistics

**Fig. 3:**
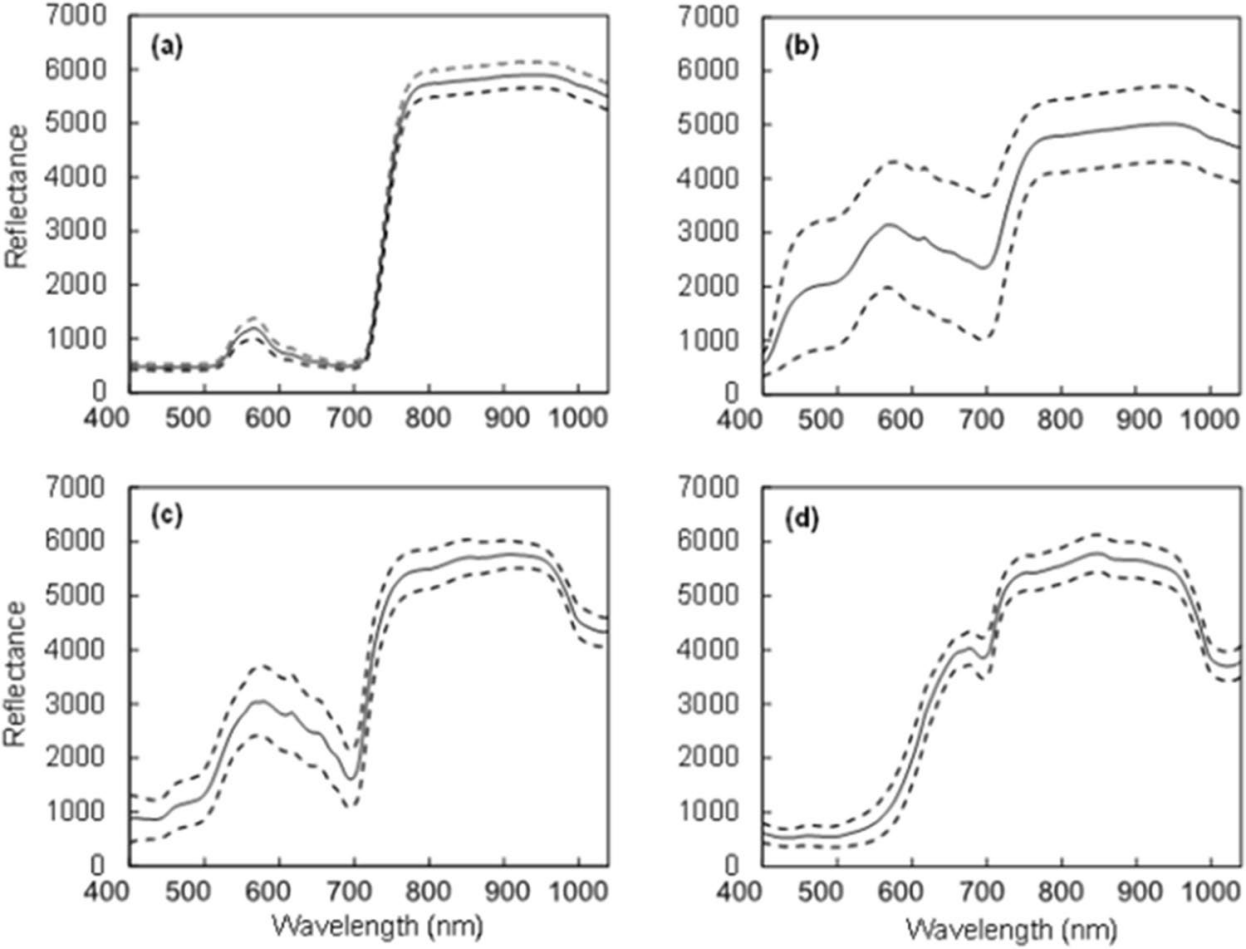
The mean (±SD) corrected relative reflectance of the Vis/NIR spectrum (400–1,000 nm) from strawberry **a** fresh leaves (*n* = 300), **b** fresh flowers (*n* = 120), **c** unripe fruit (*n* = 180), and **d** ripe fruit (*n* = 620). The 100% reflectivity was scaled to 10,000 (integers) by default

Reflectance of the Vis/NIR spectrum (400–1,000 nm) from strawberry leaves, unripe fruit, and ripe fruit had low standard deviations, while the spectra of randomly harvested flowers (with sepals) had large standard deviations (Fig. 3). The calibration and test data sets used for developing each of the models had comparable means and ranges (Table A1).

### Estimating nitrogen concentrations

**Fig. 4:**
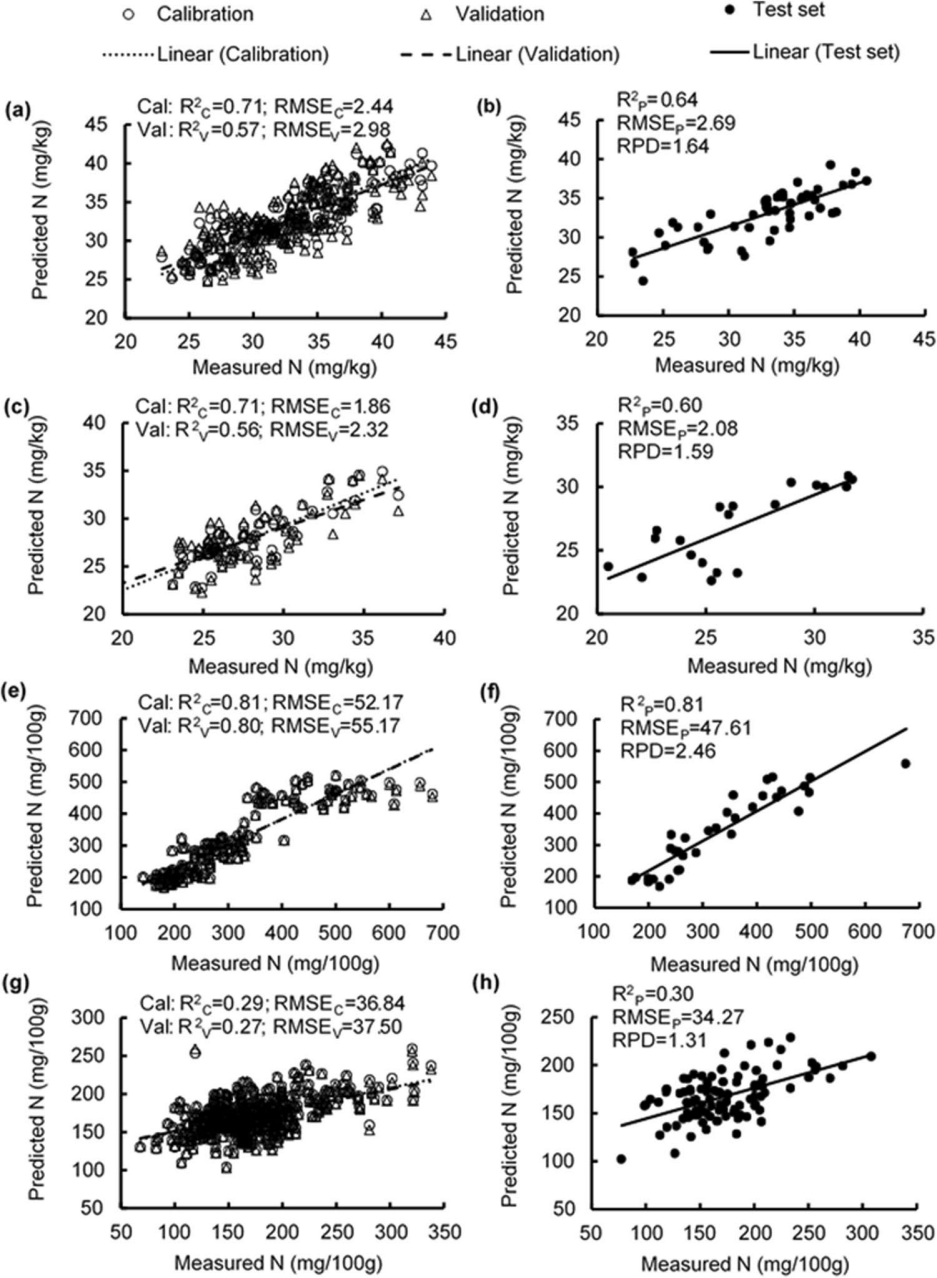
Measured vs. predicted nitrogen (N) concentration in strawberry **a, b** fresh leaves, **c, d** fresh flowers, **e, f** unripe fruit, and **g, h** ripe fruit of the calibration set (Cal, open circles), validation set (Val, open triangles), and test set (closed circles) using selected wavelengths

**Fig. 5:**
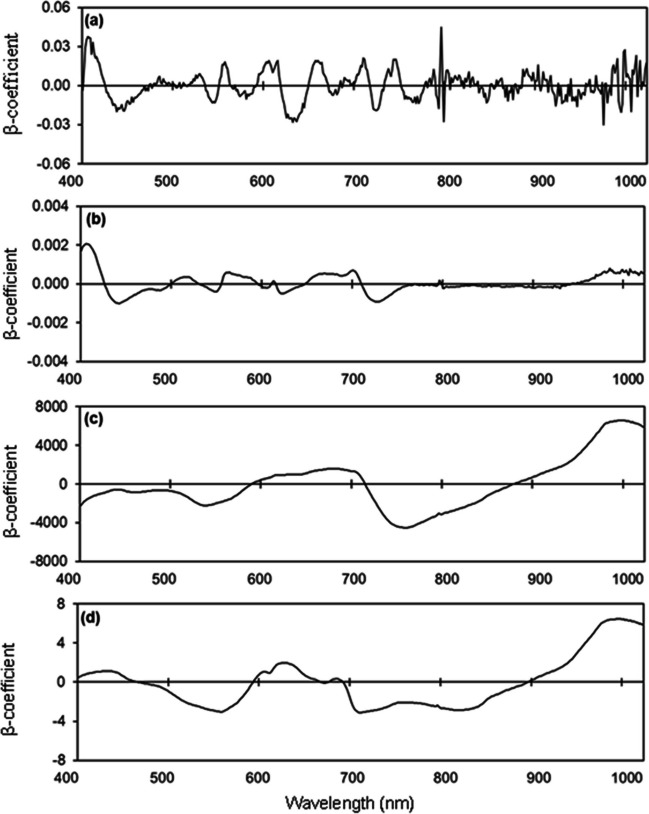
Weighted beta coefficients of predicted nitrogen (N) concentration in strawberry **a** fresh leaves, **b** fresh flowers, **c** unripe fruit, and **d** ripe fruit

The PLSR model estimated N concentration in leaves with *R*^2^ of 0.71, 0.57, and 0.64 and RMSE values of 2.44, 2.98, and 2.69 mg/kg for the calibration, validation, and test sets, respectively (Fig. 4 a, b). The RPD of foliar N prediction was 1.64 (Table A3; Fig. 4b). The *R*^2^ values in estimating flower N concentrations were 0.56–0.71, RMSE values were 1.86–2.32 mg/kg, and the RPD was 1.59 (Table A3; Fig. 4 c, d). Estimation of N concentration in unripe fruit had high accuracy, with *R*^2^ from 0.80–0.81, RMSE values from 47.61–55.17 mg/100 g, and RPD of 2.46 (Table A3; Fig. 4 e, f). In contrast, the PLSR model for estimating N concentration in ripe fruit had poor accuracy, with *R*^2^ below 0.30, RMSE values from 34.27–37.50 mg/100 g, and RPD of 1.31 (Table A3; Fig. 4 g, h). High β-coefficients were observed at 450 nm, 550 nm, 620 nm, 690 nm, and 960 nm for estimating N concentration in leaves and ripe fruit; at 420 nm, 440 nm, 540 nm, 690 nm, 720 nm, and 960 nm for flowers; and at 400 nm, 520 nm, 620 nm, 720 nm, and 960 nm for unripe fruit (Fig. 5).

### Estimating phosphorus concentrations

**Fig. 6:**
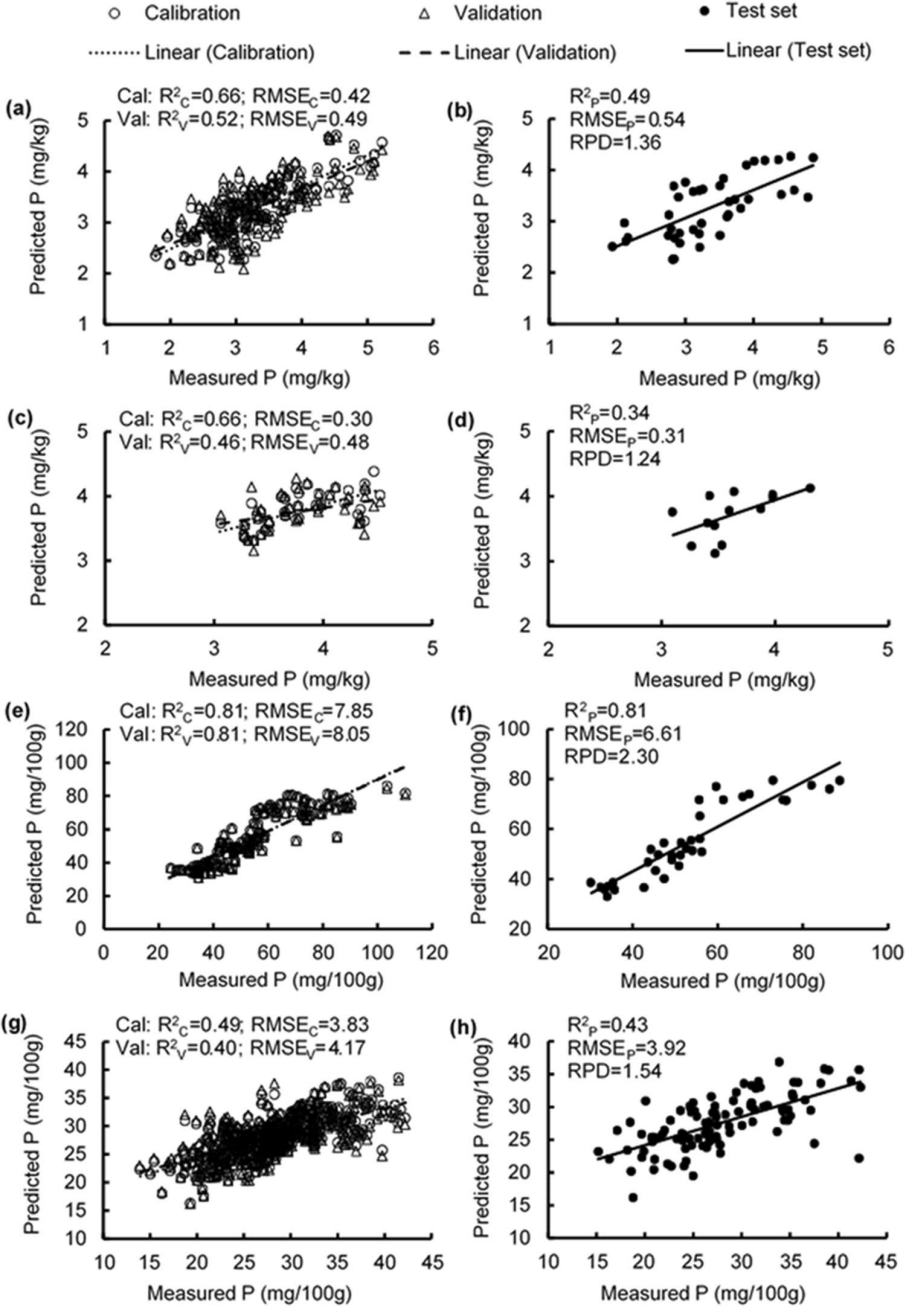
Measured vs. predicted phosphorus (P) concentration in strawberry **a, b** fresh leaves, **c, d** fresh flowers, **e, f** unripe fruit, and **g, h** ripe fruit of the calibration set (Cal, open circles), validation set (Val, open triangles), and test set (closed circles) using selected wavelengths

**Fig. 7:**
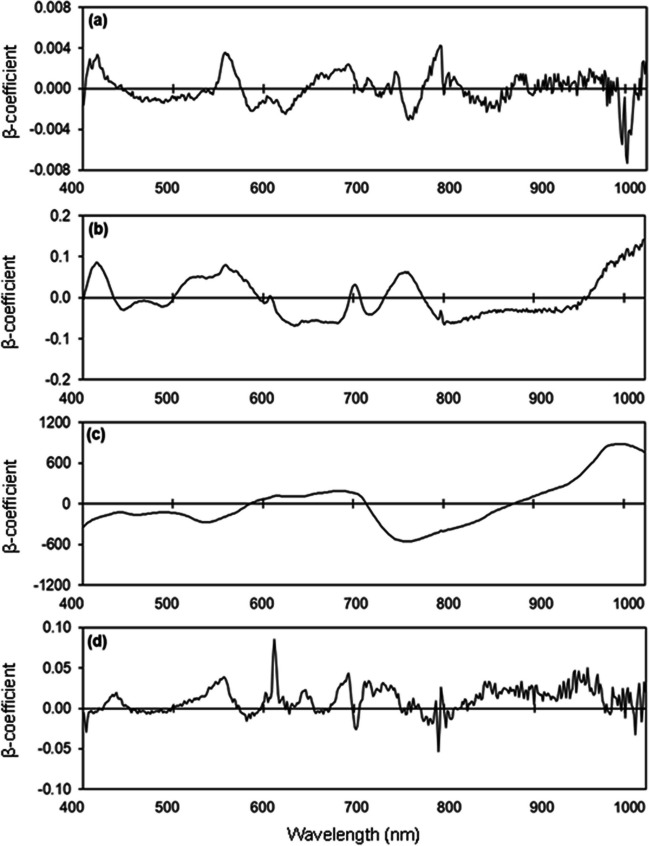
Weighted beta coefficients of predicted phosphorus (P) concentration in strawberry **a** fresh leaves, **b** fresh flowers, **c** unripe fruit, and **d** ripe fruit

The *R*^2^ values in estimating foliar P concentration were 0.49–0.66, RMSE values were 0.42–0.54 mg/kg, and the RPD value was 1.36 (Table A3; Fig. 6 a, b). Similarly, the *R*^2^ values in estimating flower P concentration were 0.34–0.66, RMSE values were 0.30–0.48 mg/kg, and the RPD was 1.24 (Table A3; Fig. 6 c, d). High estimation accuracy was obtained in estimating the P concentration of unripe fruit, with *R*^2^ of 0.81 for the calibration, validation, and test sets; RMSE values from 6.61–8.05 mg/100 g; and RPD of 2.30 (Table A3; Fig. 6 e, f). In contrast, estimation of P concentration for ripe fruit had lower accuracy, with *R*^2^ values being 0.40–0.49, RMSE values being 3.83–4.17 mg/100 g, and the RPD being 1.54 (Table A3; Fig. 6 g, h). Prominent peaks were observed in the 520 nm, 590 nm, 660 nm, 790 nm, 930 nm, and 960 nm regions in the PLSR models for estimating P concentration in leaves and ripe fruit, and in the 400 nm, 550 nm, 690 nm, 760 nm, and 960 nm regions for flowers and unripe fruit (Fig. 7).

### Estimating potassium concentrations

**Fig. 8:**
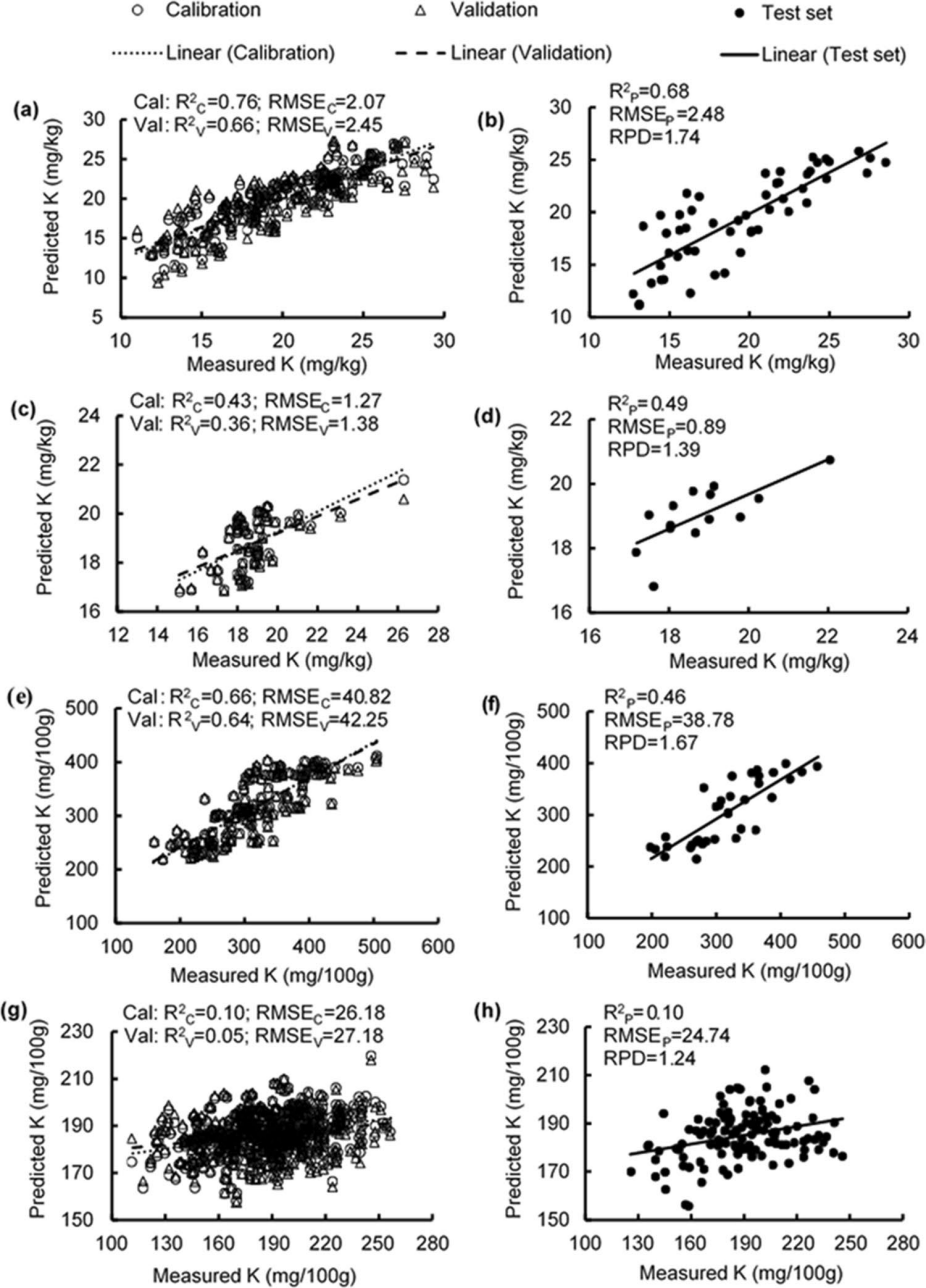
Measured vs. predicted potassium (K) concentration in strawberry **a, b** fresh leaves, **c, d** fresh flowers, **e, f** unripe fruit, and **g, h** ripe fruit of the calibration set (Cal, open circles), validation set (Val, open triangles), and test set (closed circles) using selected wavelengths

**Fig. 9:**
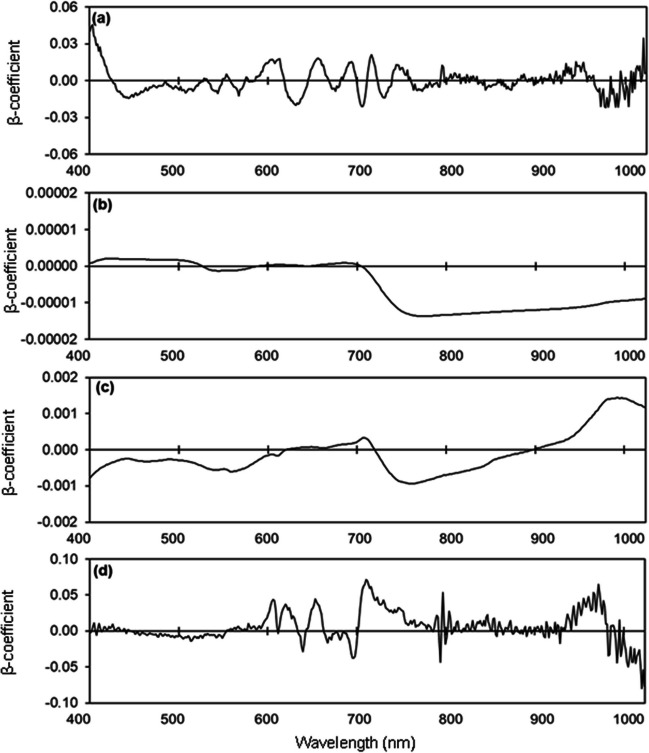
Weighted beta coefficients of predicted potassium (K) concentration in strawberry **a** fresh leaves, **b** fresh flowers, **c** unripe fruit, and **d** ripe fruit

The *R*^2^ values in estimating leaf K concentration were 0.66–0.76, RMSE values were from 2.07–2.48 mg/kg, and the RPD was 1.74 (Table A3; Fig. 8 a, b). The PLSR models to estimate K concentration in flowers had *R*^2^ values from 0.36–0.49, RMSE values from 0.89–1.38 mg/kg, and RPD of 1.39 (Table A3; Fig. 8 c, d). The *R*^2^ values in estimating K concentration of unripe fruit were 0.46–0.66, RMSE values were 38.78–42.25 mg/100 g, and RPD was 1.67 (Table A3; Fig. 8 e, f). However, low estimation accuracy was obtained in predicting the K concentration of ripe fruit, with *R*^2^ values being 0.05–0.10 (Fig. 8 g, h). Weighted β-coefficients with high peaks were observed in the 400 nm, 750 nm, 960 nm, and 990 nm regions in the developed models for estimating K concentration in leaves, flowers, unripe fruit, and ripe fruit (Fig. 9).

### Estimating calcium concentrations

**Fig. 10:**
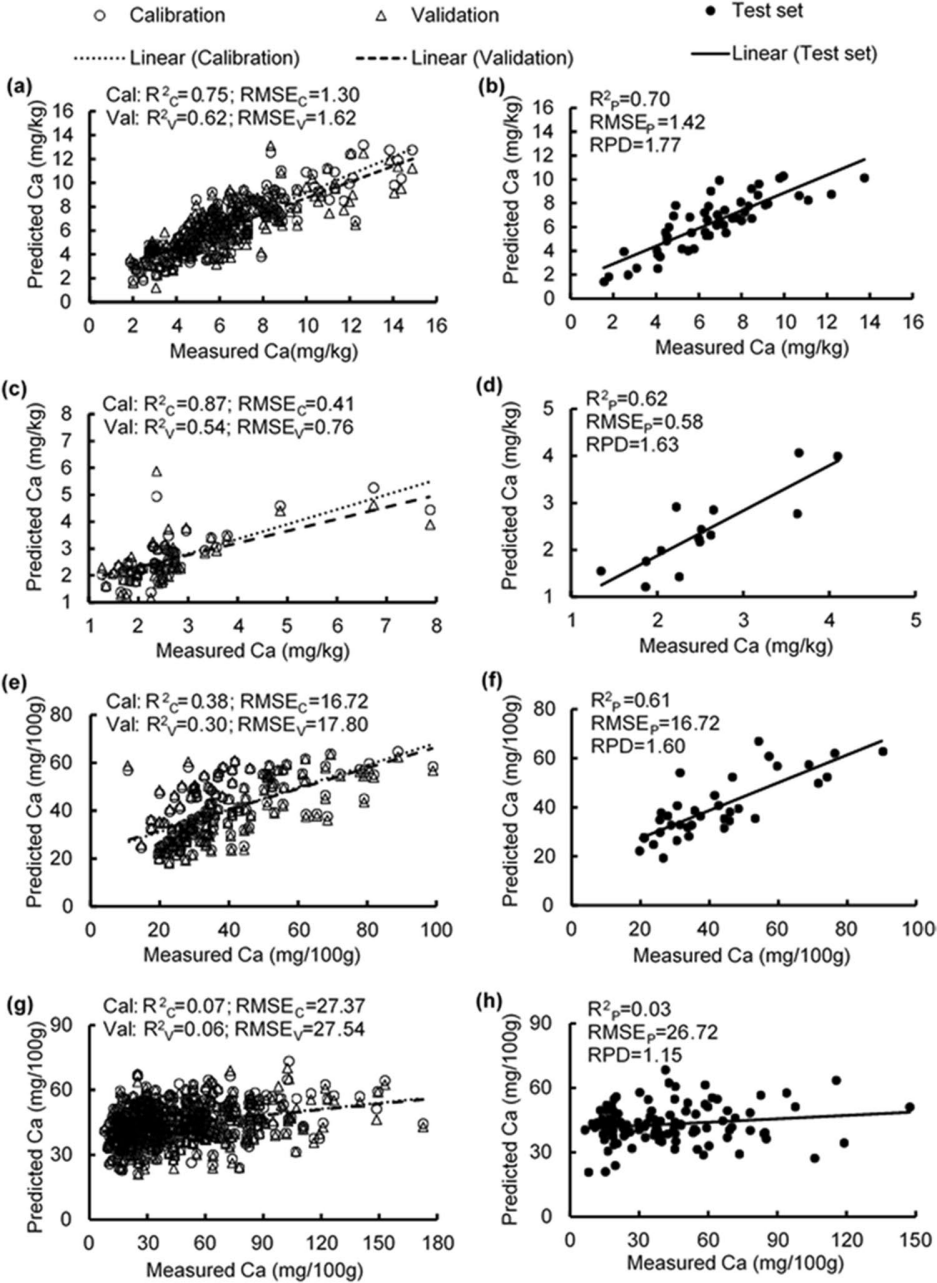
Measured vs. predicted calcium (Ca) concentration in strawberry **a, b** fresh leaves, **c, d** fresh flowers, **e, f** unripe fruit, and **g, h** ripe fruit of the calibration set (Cal, open circles), validation set (Val, open triangles), and test set (closed circles) using selected wavelengths

**Fig. 11:**
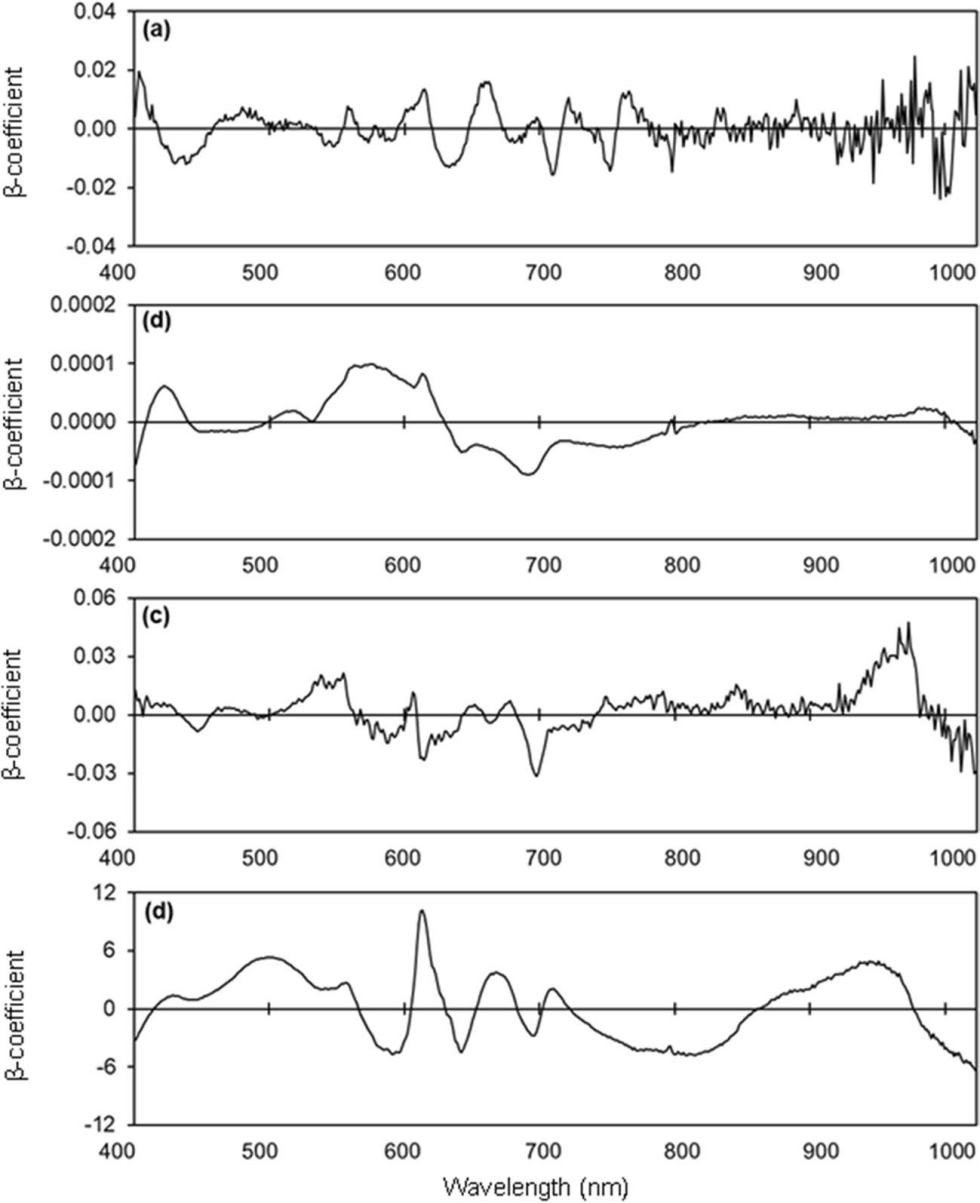
Weighted beta coefficients of predicted calcium (Ca) concentration in strawberry **a** fresh leaves, **b** fresh flowers, **c** unripe fruit, and **d** ripe fruit

The PLSR model estimated leaf Ca concentration with *R*^2^ values of 0.75, 0.62, and 0.70 and RMSE values of 1.30, 1.62, and 1.42 mg/kg for the calibration, validation, and test set, respectively, and RPD of 1.77 (Table A3; Fig. 10 a, b). The model for estimating Ca concentration of flowers had *R*^2^ values from 0.54–0.87, RMSE values from 0.41–0.76 mg/kg, and RPD of 1.63 (Table A3; Fig. 10 c, d). The model for estimating Ca concentration of unripe fruit provided *R*^2^ values from 0.30–0.61, RMSE values from 16.72–17.80 mg/100 g, and RPD of 1.60 (Table A3; Fig. 10 e, f). The model for estimating Ca concentration of ripe fruit had *R*^2^ values from 0.03–0.07, RMSE values from 26.72–27.54 mg/100 g, and RPD of 1.15 (Table A3; Fig. 10 g, h). High peaks were observed in the 410 nm, 420 nm, 620 nm, 650 nm, 690 nm, 920 nm, 960 nm, and 990 nm regions in the models for estimating leaf Ca concentration (Fig. 11). Prominent peaks were observed in the 400 nm, 420 nm, 550 nm, 660 nm, and 990 nm regions for fresh flowers, in the 400 nm, 550 nm, 590 nm, 690 nm, 920 nm, and 990 nm regions for unripe fruit, and in the 400 nm, 490 nm, 590 nm, 790 nm, 920 nm, and 990 nm regions for ripe fruit (Fig. 11).

### Predicting yield and fruit mass

Fruit yield per plant (*R*^2^ = 0.256) and fruit mass (*R*^2^ = 0.226) had significant linear relationships with the Difference Vegetation Index, DVI _[800, 670]_, based on the leaf reflectance spectrum (Table 1). Fruit yield per plant (*R*^2^ = 0.294) and fruit mass (*R*^2^ = 0.307) were also related significantly to leaf N concentration (Table 1). Fruit yield per plant and fruit mass were not related significantly to other vegetation indices or leaf nutrient concentrations (Table 1).

**Table 1:** Stepwise regressions to identify factors explaining fruit yield and fruit mass of strawberry using vegetation indices or leaf nutrient concentrations as independent parameters

Parameter	Vegetation indices			Leaf nutrient concentrations		
	**Independent**	***R***^**2**^	**Probability**	**Independent**	***R***^**2**^	**Probability**
Fruit yield	DVI	0.256	*p* = 0.004	Nitrogen	0.294*	*p* = 0.002
	MCARI	0.010	*p* = 0.380	Phosphorus	0.005*	*p* = 0.560
	MTVI	0.010	*p* = 0.393	Potassium	0.012*	*p* = 0.741
	NDVI	0.124	*p* = 0.191	Calcium	0.005*	*p* = 0.569
	PRI	0.011	*p* = 0.846			
	EVI	0.005	*p* = 0.303			
	RVI	0.079	*p* = 0.191			
	OSAVI	0.0002	*p* = 0.619			
	IPVI	0.0002	*p* = 0.619			
	SIPI	0.079	*p* = 0.098			
	RVSI	0.118	*p* = 0.680			
Fruit mass	DVI	0.226	*p* = 0.008	Nitrogen	0.307*	*p* = 0.001
	MCARI	0.016	*p* = 0.317	Phosphorus	0.018*	*p* = 0.833
	MTVI	0.017	*p* = 0.330	Potassium	0.011*	*p* = 0.710
	NDVI	0.100	*p* = 0.262	Calcium	0.003*	*p* = 0.650
	PRI	0.013	*p* = 0.306			
	EVI	0.001	*p* = 0.717			
	RVI	0.063	*p* = 0.388			
	OSAVI	0.001	*p* = 0.464			
	IPVI	0.001	*p* = 0.464			
	SIPI	0.076	*p* = 0.116			
	RVSI	0.123	*p* = 0.539			

An asterisk (*) indicates a negative *r* value; *DVI*, Difference Vegetation Index; *MCARI*, Modified Chlorophyll Absorption Ratio Index; *MTVI*, Modified Triangle Vegetation Index; *NDVI*, Normalized Difference Vegetation Index; *PRI*, Photochemical Reflectance Index; *EVI*, Enhanced Vegetation Index; *RVI*, Ratio Vegetation Index; *IPVI*, Infrared Percentage Vegetation Index; *SIPI*, Structure Independent Pigments Index; *RVSI*, Red Edge Vegetation Stress Index

## Discussion

Our results showed that visible and near infrared wavelengths (400–1,000 nm) had high accuracy in estimating nitrogen, phosphorus, potassium, and calcium concentrations in strawberry unripe fruit, and in estimating nitrogen, potassium and calcium concentrations in strawberry leaves and flowers. However, macronutrient concentrations in ripe fruit could not be estimated accurately. We also found that fruit yield per plant and fruit mass could be predicted using a hyperspectral-based vegetation index or leaf nitrogen concentrations. Hyperspectral imaging, therefore, has great potential for diagnosing the nutrient status of strawberry plants and providing decision support to amend fertilizer scheduling.

Nitrogen, phosphorus, potassium, and calcium concentrations in strawberry leaves, flowers, and unripe fruit were estimated with higher accuracies than in ripe fruit. The models for estimating nitrogen and phosphorus concentrations in unripe fruit provided coarse quantitative predictions, while the models for potassium and calcium concentrations in unripe fruit only provided discrimination between low and high values. Furthermore, the models for nitrogen and calcium concentrations in leaves and flowers and potassium concentrations in leaves also provided simple discrimination between low and high values. The ratio of performance to deviation (RPD) is one of the most commonly used criteria in determining model robustness for accurately predicting a variable. PLSR models can discriminate between low and high values, but not provide good quantitative predictions, when the RPD value is between 1.5 and 2.0 (Nicolaï et al. 2007). High-accuracy estimations of leaf macronutrient concentrations have been achieved in previous studies of strawberry and other crops such as cacao, citrus, corn, maize, pepper, and soybean using hyperspectral imaging, mainly under field conditions (Ercoli et al. 1993; España-Boquera et al. 2006; Pandey et al. 2017; Yanli et al. 2015; Yu et al. 2014; Zhu et al. 2006). We found that hyperspectral imaging could also be used to estimate macronutrient concentrations of strawberry flowers and unripe fruit using laboratory-based hyperspectral imaging, suggesting another potential approach for fertilizer planning.

Some mineral nutrients do not absorb light in the visible and near infrared regions and so visible and near infrared spectroscopy cannot detect these minerals directly (Manley 2014). Hyperspectral imaging can possibly detect these minerals and their concentrations indirectly, for example, when minerals bind to organic complexes (Manley 2014). Nitrogen exists in plant parts in several forms, including in amino acids, proteins, and chlorophyll molecules. Hyperspectral imaging systems that use visible and near infrared wavelengths can possibly detect nitrogen indirectly through chlorophyll-related compounds, because chlorophylls are strongly absorptive in the blue and red parts of the visible region (Ercoli et al. 1993; Pacumbaba and Beyl 2011; Pandey et al. 2017). Phosphorus is a component part of proteins and nucleic acids and so hyperspectral imaging can possibly detect phosphorus indirectly by detecting these organic macromolecules (Raven 2013). Potassium has a vital role in plant water absorption and osmotic potential regulation and accumulates as a mineral element in the vacuole, and so hyperspectral imaging may detect potassium concentrations indirectly through changes in water potential and solute concentrations that are related to potassium concentrations (Egilla et al. 2005; Malmir et al. 2020; Vago et al. 2009). Calcium may not be spectrally active, but estimation is possible when it binds with molecules that have covalent bonds such as N–H, S–H, O–H, C–H, C–O, or C = C (Bellon-Maurel et al. 2010; Manley 2014).

We were able to identify specific wavelengths important for estimating nitrogen, phosphorus, potassium, and calcium concentrations. Wavelengths with the highest β-coefficients contribute most to the predictive ability of models (Bai et al. 2018; Malmir et al. 2019). High β-coefficients at 400–450 nm, 530–550 nm, 590–650 nm, 700–780 nm, and 960–1000 nm have also been reported when estimating nitrogen, phosphorus, potassium, and calcium concentrations in cacao, pepper, and wheat leaves and avocado fruit (Hosseini-Bai et al. 2019; Kämper et al. 2020; Malmir et al. 2020; Yao et al. 2010; Yu et al. 2014). We found that prominent wavelengths for estimating nitrogen concentrations were in the regions of 400–550 nm, 620–720 nm, and 960–990 nm, which includes the blue and red regions of the visible spectrum at which chlorophyll is highly absorptive (Pandey et al. 2017). Chlorophyll concentrations are often significantly correlated with nitrogen concentrations (Bojović and Marković 2009). The wavelengths with high β-coefficients for estimating phosphorus, potassium, and calcium concentrations were in the regions of 570–600 nm, 710–730 nm, 770–820 nm, and 860–990 nm. Reflectance in the region of 460 nm or 670 nm is the result of electron transitions in chlorophyll a and b, and reflectance in the region of 950–1000 nm is associated with prominent molecular bonds such as O–H, C–H, and N–H in water, starch, and proteins (Curran 1989; Zur et al. 2000).

Strawberry fruit yield and fruit mass could be predicted using hyperspectral imaging of leaves using the Difference Vegetation Index (DVI). This index was the only vegetation parameter, among the 11 vegetation indices we examined, that had a significant linear relationship with yield or mass. Grain yield can also be predicted using DVI based on hyperspectral canopy reflectance (350–2,500 nm), but with higher *r*^2^ values of 0.77–0.81 (Cao et al. 2015) compared with < 0.30 in our study. PLSR usually outperforms other deep learning models when small datasets are used, although deep learning techniques can be applied to increase the prediction accuracy when a larger dataset exists (Ludwig et al. 2019).

Strawberry yield was also predicted from foliar nitrogen concentrations, but not phosphorus, potassium, or calcium concentrations. Plant yield often has strong positive relationships with foliar nitrogen and chlorophyll concentrations (Reis et al. 2009). However, leaf nitrogen concentrations had a significant negative relationship (*r* =  − 0.54) with yield in our study and in studies of apple (*r* =  − 0.82) and green pepper (*r* =  − 0.69) (Drake et al. 2002; Hassan et al. 1993). Nitrogen excess can reduce strawberry yield and fruit size (Trejo-Téllez and Gómez-Merino 2014). Highest yields of Tudla strawberry correspond to foliar nitrogen concentrations of 2.1–3.0% (Drake et al. 2002). Foliar nitrogen concentrations in Redlands Joy leaves were often between 3.0 and 4.5% (Dung et al. 2022), which was higher than in Tudla strawberry, and these high concentrations might explain the negative correlation between yield and nitrogen concentration in our study. The recommended concentrations for phosphorus, potassium, and calcium in Tudla strawberry leaves range from 0.20–0.38%, 1.84–2.21%, and 0.77–1.48%, respectively (Almaliotis et al. 2002). The concentrations of phosphorus, potassium, and calcium in our study were within these ranges (Dung et al. 2022), although there are no published recommendations for nutrient levels in Redlands Joy strawberry plants.

## Conclusion

Laboratory-based hyperspectral imaging showed great potential for estimating nitrogen, phosphorus, potassium, and calcium concentrations in strawberry plants, with often-high estimation accuracies for leaves, flowers, and unripe fruit. The technology also showed potential for predicting yield and fruit mass using the Difference Vegetation Index. Hyperspectral imaging may, therefore, be used by strawberry growers to monitor plant nutrient status and manage fertilizer inputs in real time during flowering and fruit growth to ensure the best possible fruit yield and quality. Prediction of nitrogen, phosphorus, potassium, and calcium concentrations using hyperspectral imaging of ripe fruit will require further investigation using deep learning techniques.

### Supplementary Information

Below is the link to the electronic supplementary material.

Supplementary file1 (DOCX 1325 KB)

## Data Availability

Data will be made available upon the request.
