# Supplementary material for: Hyperspectral imaging for estimating leaf, flower, and fruit macronutrient concentrations and predicting strawberry yields
Source: Environ Sci Pollut Res Int. 2023 Oct 19;30(53):114166–82. doi: 10.1007/s11356-023-30344-8 (PMC10663281; doi:10.1007/s11356-023-30344-8)
Supplement: Supplementary file 1 — Supplementary file1 (DOCX 1325 KB) [file 11356_2023_30344_MOESM1_ESM.docx]

**Supplementary Information**


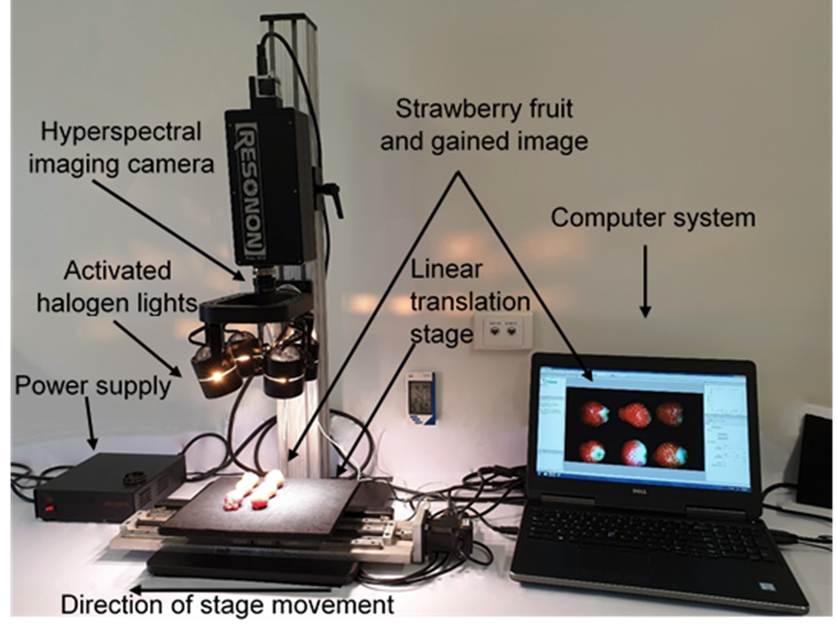


**Fig.** **A1.** The hyperspectral imaging system used in the study


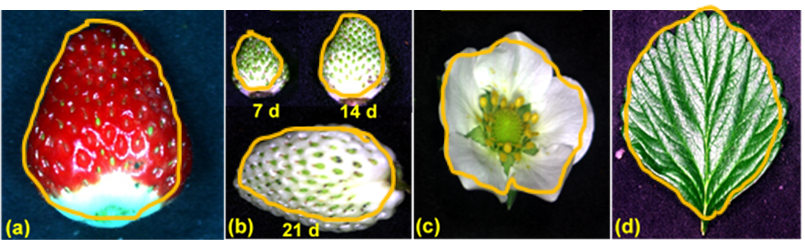


**Fig. A2.** The region of interest (ROI), shown in yellow circles, for strawberry **(a)** ripe fruit, **(b)** unripe fruit harvested at 7, 14 or 21 d after first pollination, **(c)** flower and **(d)** leaf used for developing models to estimate nitrogen, phosphorus, potassium and calcium concentrations.

| **Table A1** Descriptive statistics for calibration and test data sets (sample number, minimum and maximum values, range, CV*, mean and SD*) for nitrogen, phosphorus, potassium and calcium concentrations in strawberry leaves, flowers, unripe fruit and ripe fruit using all wavelength bands. | | | | | | | | | | | | | | | | |
| --- | --- | --- | --- | --- | --- | --- | --- | --- | --- | --- | --- | --- | --- | --- | --- | --- |
|  |  |  |  |  |  |  |  |  |  |  |  |  |  |  |  |  |
| **Plant part** | **Set** | **n** | **Min.** | **Max.** | **Range** | **CV** | **Mean** | **SD** |  | **n** | **Min.** | **Max.** | **Range** | **CV** | **Mean** | **SD** |
|  |  | **Nitrogen** | | | | | | |  | **Phosphorus** | | | | | | |
| Fresh leaf  (mg/kg) | Calibration | 210 | 24.83 | 43.95 | 19.12 | 0.14 | 32.47 | 4.56 |  | 160 | 2.26 | 5.23 | 2.97 | 0.21 | 3.26 | 0.68 |
|  | Test | 52 | 24.43 | 40.54 | 16.11 | 0.14 | 32.80 | 4.46 |  | 40 | 2.26 | 4.88 | 2.62 | 0.22 | 3.36 | 0.74 |
| Fresh flower  (mg/kg) | Calibration | 70 | 21.77 | 37.18 | 14.41 | 0.12 | 27.73 | 3.41 |  | 42 | 3.31 | 4.51 | 1.20 | 0.141 | 3.78 | 0.40 |
|  | Test | 20 | 22.60 | 31.74 | 9.14 | 0.13 | 26.43 | 3.38 |  | 13 | 3.12 | 4.31 | 1.19 | 0.09 | 3.62 | 0.34 |
| Unripe fruit  (mg/100 g) | Calibration | 139 | 168.45 | 678.96 | 510.419 | 0.39 | 317.0 | 122.17 |  | 131 | 33.51 | 110.07 | 76.56 | 0.33 | 54.76 | 18.82 |
|  | Test | 35 | 167.76 | 675.71 | 507.95 | 0.37 | 323.32 | 118.70 |  | 36 | 33.00 | 88.63 | 55.63 | 0.29 | 53.37 | 15.39 |
| Ripe fruit  (mg/100 g) | Calibration | 400 | 103.93 | 336.85 | 232.92 | 0.26 | 170.32 | 43.81 |  | 402 | 16.30 | 42.23 | 25.93 | 0.19 | 27.93 | 5.36 |
|  | Test | 100 | 102.18 | 307.70 | 205.52 | 0.24 | 171.90 | 40.82 |  | 99 | 16.17 | 42.14 | 25.97 | 0.22 | 27.70 | 6.07 |
|  |  | **Potassium** | | | | | | |  | **Calcium** | | | | | | |
| Fresh leaf  (mg/kg) | Calibration | 198 | 11.18 | 29.31 | 18.13 | 0.21 | 19.91 | 4.15 |  | 210 | 1.79 | 14.90 | 13.11 | 0.42 | 6.24 | 2.62 |
|  | Test | 52 | 11.13 | 28.56 | 17.43 | 0.22 | 19.39 | 4.35 |  | 52 | 1.41 | 13.75 | 12.34 | 0.38 | 6.65 | 2.55 |
| Fresh flower  (mg/kg) | Calibration | 55 | 16.81 | 26.24 | 9.43 | 0.09 | 18.80 | 1.69 |  | 54 | 1.32 | 7.85 | 6.53 | 0.44 | 2.57 | 1.13 |
|  | Test | 14 | 16.81 | 22.06 | 5.25 | 0.07 | 18.79 | 1.28 |  | 14 | 1.21 | 4.11 | 2.90 | 0.30 | 2.55 | 0.77 |
| Unripe fruit  (mg/100 g) | Calibration | 143 | 217.81 | 504.61 | 286.80 | 0.22 | 315.82 | 70.39 |  | 135 | 19.63 | 98.73 | 79.10 | 0.43 | 38.55 | 16.74 |
|  | Test | 35 | 214.51 | 458.15 | 243.64 | 0.21 | 315.78 | 65.52 |  | 36 | 19.31 | 90.00 | 70.69 | 0.42 | 41.99 | 17.64 |
| Ripe fruit  (mg/100 g) | Calibration | 480 | 158.07 | 256.62 | 98.55 | 0.15 | 186.05 | 27.68 |  | 437 | 21.3 | 172.73 | 151.44 | 0.64 | 44.18 | 28.35 |
|  | Test | 120 | 155.72 | 245.81 | 90.09 | 0.14 | 188.92 | 25.70 |  | 110 | 20.6 | 147.36 | 126.76 | 0.65 | 41.51 | 26.82 |
| * CV: coefficient of variation; Min.: minimum; Max. maximum; SD: standard deviation. | | | | | | | | | | | | | | | | |

| **Table A2** Equations for spectral indices used in the study | | |
| --- | --- | --- |
| **Vegetation**  **index** | **Algorithm** | **Reference** |
| DVI | R800 – R670 | Rathod et al. 2013 |
| MCARI | [(R700-R670) - 0.2 × (R700-R670)] × (R700 ÷ R670) | Wang et al. 2018 |
| MTVI | 1.5 × [1.2 × (R710 - R550) - 2.1 × (R670 - R550)] | Rathod et al. 2013 |
| NDVI | (R800 - R670) ÷ (R800 + R670) | Wang et al. 2018 |
| PRI | (R530 - R570) ÷ (R530 + R570) | Rathod et al. 2013 |
| EVI | 2.5 × [(R840 – R660) ÷ (R840 + 6 × R660 – 7.5 × R490 + 1)] | Yu et al. 2018 |
| RVI | (R800 ÷ R670) | Rathod et al. 2013 |
| IPVI | R800 ÷ (R800 + R670) | Rathod et al. 2013 |
| SIPI | (R810 - R460) ÷ (R810 - R680) | Wang et al. 2018 |
| RVSI | (R710 + R750) ÷ 2 – R730) | Wang et al. 2018 |
| Note: R denotes reflectance spectrum; DVI = Difference Vegetation Index; MCARI = Modified Chlorophyll Absorption Ratio Index; MTVI = Modified Triangle Vegetation Index; NDVI = Normalized Difference Vegetation Index; PRI = Photochemical Reflectance Index; EVI = Enhanced Vegetation Index; RVI = Ratio Vegetation Index; IPVI = Infrared Percentage Vegetation Index; SIPI = Structure Independent Pigments Index and RVSI = Red Edge Vegetation Stress Index. | | |

| **Table A3** Performance of developed partial least squares regression models in predicting nitrogen, phosphorus, potassium and calcium concentrations in strawberry fresh leaves (mg/kg), fresh flowers (mg/kg), unripe fruit (mg/100 g) and ripe fruit (mg/100 g) using selected wavelengths | | | | | | | |
| --- | --- | --- | --- | --- | --- | --- | --- |
| **Plant part** | **Data**  **pre-processing method** | **LV** | **Calibration set** | | **Validation set** | | **RPD** |
|  |  |  | **RMSE_C_ R^2^_C_** | | **RMSE_V_ R^2^_V_** | |  |
| Nitrogen | | | | | | | |
| Fresh leaf | Raw data | 15 | 2.44 | 0.71 | 2.98 | 0.57 | 1.64 |
| Fresh flower | Raw data | 7 | 1.86 | 0.71 | 2.32 | 0.56 | 1.59 |
| Unripe fruit | Normalize | 2 | 52.17 | 0.81 | 55.17 | 0.80 | 2.46 |
| Ripe fruit | SNV | 2 | 36.84 | 0.29 | 37.50 | 0.27 | 1.31 |
| Phosphorus | | | | | | | |
| Fresh leaf | Raw data | 14 | 0.42 | 0.66 | 0.49 | 0.52 | 1.36 |
| Fresh flower | SNV | 6 | 0.30 | 0.66 | 0.48 | 0.46 | 1.24 |
| Unripe fruit | Normalize | 2 | 7.85 | 0.81 | 8.05 | 0.81 | 2.30 |
| Ripe fruit | DSG | 8 | 3.83 | 0.49 | 4.17 | 0.40 | 1.54 |
| Potassium | | | | | | | |
| Fresh leaf | Raw data | 15 | 2.07 | 0.76 | 2.45 | 0.66 | 1.74 |
| Fresh flower | Raw data | 2 | 1.27 | 0.43 | 1.38 | 0.36 | 1.39 |
| Unripe fruit | Raw data | 3 | 40.82 | 0.66 | 42.25 | 0.64 | 1.67 |
| Ripe fruit | DSG | 4 | 26.18 | 0.10 | 27.18 | 0.05 | 1.24 |
| Calcium | | | | | | | |
| Fresh leaf | Raw data | 15 | 1.30 | 0.75 | 1.62 | 0.62 | 1.77 |
| Fresh flower | SNV | 14 | 0.41 | 0.87 | 0.76 | 0.54 | 1.63 |
| Unripe fruit | DSG | 6 | 16.72 | 0.38 | 17.80 | 0.30 | 1.60 |
| Ripe fruit | Raw data | 1 | 27.37 | 0.07 | 27.54 | 0.06 | 1.15 |
| LV: Number of latent variables; RPD: ratio of performance to deviation; RMSE_C_: root mean squares error of calibration, RMSE_V_: root mean squares error of validation; R2C: determination coefficient of calibration; R2V: determination coefficient of validation; SNV: Standard Normal Variate; DSG: Derivative S-Golay | | | | | | | |
